# Regulation of γδ T Cell Effector Diversification in the Thymus

**DOI:** 10.3389/fimmu.2020.00042

**Published:** 2020-01-24

**Authors:** Morgan E. Parker, Maria Ciofani

**Affiliations:** Department of Immunology, Duke University Medical Center, Durham, NC, United States

**Keywords:** γδ T cells, thymus, TCR signal strength, transcriptional regulation, innate-like lymphocyte, IL-17A, IFNγ

## Abstract

γδ T cells are the first T cell lineage to develop in the thymus and take up residence in a wide variety of tissues where they can provide fast, innate-like sources of effector cytokines for barrier defense. In contrast to conventional αβ T cells that egress the thymus as naïve cells, γδ T cells can be programmed for effector function during development in the thymus. Understanding the molecular mechanisms that determine γδ T cell effector fate is of great interest due to the wide-spread tissue distribution of γδ T cells and their roles in pathogen clearance, immunosurveillance, cancer, and autoimmune diseases. In this review, we will integrate the current understanding of the role of the T cell receptor, environmental signals, and transcription factor networks in controlling mouse innate-like γδ T cell effector commitment.

## Introduction

γδ T cells are part of the three evolutionary conserved lymphocyte lineages (with αβ T cells and B cells) that undergo somatic gene rearrangement for the generation of antigen receptors (1). While immune cells can broadly be divided by adaptive vs. innate, γδ T cells straddle this classification by having properties of both. Although γδ T cells are capable of generating unique T cell receptors (TCRs), many γδ T cells express TCRs with limited diversity (2). Innate-like γδ T cells, also referred to as “natural” γδ T cells, are endowed with their effector functions early during development in the thymus and consequently do not require clonal expansion or differentiation from a naïve cell for their effector responses (3, 4). Importantly, innate-like γδ T cells exhibit the four hallmark characteristics of tissue-resident lymphocytes; (1) self-renewal and long-term maintenance, (2) enrichment at barrier tissues, (3) tissue sensing capabilities, and (4) rapid effector responses (5). These tissue-resident properties combined with early seeding during fetal life enable innate-like γδ T cells to act as a first line of defense in the skin, gut, and reproductive tract while other lymphocytes are still being developed.

γδ T cells play innumerable roles in pathogen clearance, wound healing, autoimmunity, and cancer, largely through the production of soluble mediators (6). The two major effector subsets of γδ T cells can be distinguished based on cytokine production: IFNγ producers (Tγδ1) and IL-17A producers (Tγδ17), although γδ T cells are capable of producing many other cytokines (6). IFNγ production by γδ T cells is associated with clearance of intracellular pathogens and anti-tumor responses, while IL-17A production is linked to clearance of extracellular bacteria and fungi (7, 8). Although protective against infectious diseases, cytokine production by γδ T cells is involved in many immune pathologies and autoimmune diseases when dysregulated (9). Remarkably, the presence of γδ T cells within tumors was found to be the most significant favorable cancer-wide prognostic population in humans (10). While enriched at mucosal and barrier tissues, γδ T cells are also present in many other non-lymphoid tissues where they support steady-state tissue homeostasis (6, 11). Recent studies have shown that IL-17A production by γδ T cells regulates adipose tissue immune cell homeostasis and thermogenesis (12), bone regeneration (13), and the promotion of short-term memory in the brain meninges (14). As innate-like lymphocytes, γδ T cells sense their local environment and are regulated through a combination of the TCR, cytokine receptors, co-stimulatory receptors, inhibitory receptors, and natural killer receptors (15). These receptors recognize various environmental ligands or stimuli that induce signaling cascades that lead to expression of key transcription factors (TFs) that can then dictate the identity and effector function of γδ T cells. This review will focus on the integration of TCR and environmental cues with downstream TF modules that govern the effector fate of mouse innate-like γδ T cells.

## γδ Lineage Commitment in the Thymus

In the thymus, double-negative CD4^−^ CD8^−^ (DN) thymocytes give rise to two distinct T cell lineages defined by the expression of either an αβTCR or a γδTCR (16). DN thymocytes are a heterogeneous group of developmentally linked progenitor cells distinguished by the expression of CD44, CD117 (also known as c-kit), and CD25 that encompass the transition of early thymocyte progenitor cells (ETP/DN1) through the DN2, DN3, and DN4 cell stages (16). Rearrangement of the TCRβ, TCRγ, and TCRδ gene loci begin in DN2 cells and are completed in DN3 cells (17), a time frame that coincides with the divergence of the αβ and γδ lineages (18, 19). Indeed, the DN3 stage represents an obligatory checkpoint at which productive rearrangement and expression of either a pre-TCR (TCRβ + invariant pTα) or γδTCR complex signals the rescue of cells from apoptosis, proliferation, and αβ or γδ lineage differentiation (17). β-selected cells undergo further development to the CD4^+^CD8^+^ double positive (DP) stage, where TCRα rearrangement and additional selection events yield mature CD4^+^ or CD8^+^ single positive αβ T cells (16, 20). Unlike αβ T cells, γδ T cells develop following a single γδ-selection step mediated by the γδTCR, do not progress through to a DP stage, and rather most γδ T cells remain DN instead (16).

Developing DN thymocytes integrate signals from the TCR complex expressed on their cell surface along with myriad environmental cues. As such, two models were proposed to explain αβ vs. γδ lineage choice: the signal strength model and the stochastic-selective (pre-commitment) model (16). The major difference between these models is the importance placed on TCR signaling and the timing of its influence. The pre-commitment model is founded on the idea that lineage fate is determined prior to rearrangement of TCR loci. The expression of γδTCR on γδ T cell precursors or pre-TCR on αβ precursors simply confirms their fate and cells pre-committed to one fate with a mismatched TCR were hypothesized to die. Initial studies supporting this model showed that DN thymocytes lacking TCR expression but expressing high levels of IL-7Rα (21) or the high mobility group (HMG) box TF Sox13 (22) were predisposed to becoming γδ T cells. However, more recent evidence that Sox13 is not required for the generation of all γδ T cells, but rather only for a select subset of IL-17-producing γδ T cells marked by Vγ4 usage (23) [Tonegawa nomenclature (24)], is at odds with the pre-commitment model.

In contrast, the signal strength model of αβ vs. γδ lineage commitment has garnered widespread support. It posits that the strength of TCR signal that DN thymocytes receive dictates the lineage decision; weak signals promote αβ fate, while strong signals promote the γδ fate. The extensive evidence in favor of this model has been previously reviewed in detail (16, 25). Most notably, key support was provided by elegant experiments demonstrating that a single γδTCR transgene can mediate both γδ and αβ lineage fates, dependent on the signal strength of the TCR (26, 27). In particular, lineage fate toggled between αβ and γδ outcomes when TCR signal strength was tuned by genetic alterations in TCR ligand availability, TCR surface expression levels, or in expression of TCR signaling factors (26, 27). Enhanced or prolonged activation of the extracellular signal-regulated kinase (ERK) pathway and downstream Egr, and Id3 targets are important mediators of strong γδTCR signals that promote γδ lineage commitment (25, 26, 28). More recent work has begun to shed light on the mechanism by which DN cells translate differences in signal strength and ERK signaling into alternative lineage fates. γδ T cell development is dependent on a non-canonical mode of ERK action mediated by its DEF-binding pocket (29). This domain is favored by strong and more prolonged signals and enables ERK to bind a distinct set of proteins required for γδ lineage adoption. Thus, strong signals mediated primarily by γδTCR complexes are required for DN cell commitment to the γδ T cell lineage.

## Effector Programming of γδ T Cells

### Waves of γδ T Cell Development

A distinctive and poorly understood feature of γδ T cell ontogeny is the development of γδ thymocytes in a series of “waves” that are defined by γ-chain variable regions (Vγ) usage (Table 1). Interestingly, the waves of Vγ subsets are highly correlated with homing abilities to specific tissues early in life, where they become long-lived tissue-resident cells. This process begins when the fetal thymus is seeded as early as embryonic day 13.5 (E13.5) by fetal liver progenitors to generate the first wave of γδ T cells, known as Vγ5^+^Vδ1^+^ dendritic epidermal T cells (DETCs) that exclusively home to the epidermis of the skin (30). The second wave of γδ T cells, expressing an invariant Vγ6Vδ1 TCR, develop around E16 and primarily seed epithelial layers of the female reproductive tract, lung, and tongue (31). Next, the late fetal stages give rise to Vγ4^+^ and Vγ1^+^ γδ T cells that express more varied TCRs due to pairing with several Vδ chains and can be found in many tissues such as peripheral lymphoid organs, blood, lung, liver, and dermis (2, 31). Unlike Vγ5^+^ and Vγ6^+^ γδ T cells, these subsets are not restricted to the fetal window and can also develop during neonatal and adult life (2, 31). Of note, the Vγ7^+^ γδ T cells that reside in the intraepithelial layer of the small intestine are thought to mature extrathymically (2, 32). While the link between Vγ usage and tissue homing can be explained in DETCs with upregulation of CCR10 in the thymus before trafficking to the epidermis (33, 34), this association is not yet understood for other Vγ subsets. Moreover, the molecular mechanisms governing the unique sequential development of Vγ subsets are unknown, however features of both the fetal progenitors and environment have been implicated (35–38).

**Table 1:** Waves of γδ T cell development.

**Subset**	**V(D)J diversity**	**Timing of development**	**Tissue residence**	**Major cytokines produced**
Vγ1	High (NKT γδ T cells = Vγ1^+^Vδ6.3^+^)	Perinatal and adult	Liver, lymphoid tissues	IFNγ (IFNγ and IL-4)
Vγ4	Variable	E18 to adult	Dermis, lung, liver, lymphoid tissue	IL-17A or IFNγ
Vγ5	Invariant (Vγ5^+^Vδ1^+^)	E13-E16	Epidermis	IFNγ
Vγ6	Invariant (Vγ6^+^Vδ1^+^)	E16-birth	Uterus, lung, tongue, liver, placenta, kidney	IL-17A
Vγ7	Intermediate	Neonatal	Epithelial layer of small intestine	IFNγ

*E, embryonic day*.

### Effector Diversification of γδ Thymocytes

In contrast to αβ T cells that leave the thymus as naïve cells and acquire their effector function in the periphery, γδ T cells can commit to an effector fate during development in the thymus. The pre-programming in the thymus allows γδ T cells to be early innate-like responders to infection and tissue-damage, without the delay that is required for αβ T cell responses. While this review focuses on “pre-programmed” innate-like or “natural” γδ T cells, some γδ T cells exit the thymus as naïve cells and acquire effector function following activation in the periphery; these are referred to as “inducible” γδ T cells (4, 39). Similar to αβ T cells, innate lymphoid cells (ILCs), and other lymphocyte lineages, γδ T cells can be divided into effector subsets based on the expression of either T-bet/IFNγ (Tγδ1) or RORγt/IL-17A (Tγδ17). During ontogeny, effector γδ T cell subsets differentiate in functional waves encompassing DETCs, IL-17A producers, and NKT γδ T cells, which are also partially associated with Vγ usage (40). Specifically, Vγ5^+^ DETCs preferentially produce IFNγ, while Vγ6^+^ γδ T cells mainly produce IL-17A (41). Later waves, such as Vγ4 and Vγ1, are more heterogenous in their capacity to produce various effector cytokines. While IL-17A production is not limited to a specific Vγ subset, innate-like Tγδ17 cell generation is restricted to a window of time during fetal life, approximately E16 to birth, that enriches for Vγ6^+^ and Vγ4^+^ γδ T cell subsets (42). Within the third functional wave, Vγ1^+^Vδ6.3^+^ NKT γδ T cells express PLZF and are capable of producing both IL-4 and IFNγ (43, 44). Therefore, the fate decisions of developing thymocytes during fetal life impacts the adult reservoir of innate-like γδ T cell effectors.

**Figure 1:**
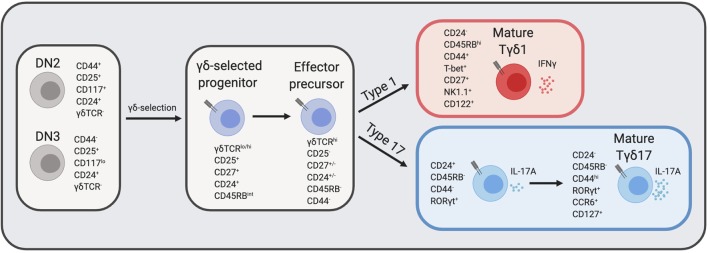
γδ T cell development in the thymus. DN thymocytes undergo γδ-selection and become immature γδ thymocytes that eventually diverge into either IFNγ producers or IL-17A producers. The expression of cell surface markers and transcription factors that define transitional precursors and mature effector γδ T cells are listed next to each cell type. CD24 and CD27 expression at the “effector precursor” stage is heterogenous and is marked by +/–, however, cells transition from CD24^+^ to CD24^−^. DN, double negative; TCR, T cell receptor. Figure made with biorender.com.

γδ T cell effectors can be defined by various cell surface markers: IFNγ producing γδ T cells typically express CD27, CD122, NK1.1, and high levels of CD45RB, while IL-17A producing γδ T cells lack expression of CD27, CD122, and NK1.1 but usually express CCR6 and low levels of CD45RB (41, 45, 46) (Figure 1). Nevertheless, the study of γδ effector diversification has been hampered by the lack of definitive markers that distinguish Tγδ1 and Tγδ17 precursors. Before effector commitment, CD25 is expressed by the earliest γδ T cells in the thymus (47), as γδ-selected thymocytes are derived from CD25^+^ DN2 and DN3 T cell precursors (18, 48). Post-selection γδ thymocytes are also distinguished by CD27 upregulation (48), and these CD25^+^CD27^+^ are the earliest progenitors of IL-17A and IFNγ γδ effectors (46). Emerging γδ thymocytes with low levels of γδTCR also express intermediate levels of CD45RB, and have molecular signatures and developmental potential consistent with being precursors to both Tγδ17 and Tγδ1 cells (41, 49). Indicative of their immature status, these pioneer γδ T cells are marked by high levels of CD24 expression, which is later downregulated upon maturation (50).

Several recent studies have provided clarity regarding the developmental trajectories of innate-like γδ T cell effector subsets beyond the precursor stage (49, 51). Recent work by Sumaria and colleagues identified CD45RB^−^CD44^−^ γδ thymocytes as precursors of both type 1 and type 17 effectors, suggesting that all γδ T cells downregulate CD45RB prior to effector diversification (Figure 1) (52). Consistent with this view, the absolute block in Tγδ17 development in the absence of c-Maf revealed an effector specialization checkpoint at the immature CD45RB^−^CD24^+^ γδ thymocyte stage (49). This block also provides genetic support for a model in which effector programming is molecularly distinct from γδ-selection (3). Among mature CD24^−^ γδ thymocytes, CD45RB and CD44 distinguish effector lineages: CD44^hi^CD45RB^lo^ γδ T express high levels of RORγt and IL-7Rα and are committed to IL-17A production, whereas CD44^+^CD45RB^+^ γδ T cells express T-bet, but lack RORγt or IL-7Rα expression and are committed to IFNγ production (Figure 1) (51). Additionally, CD73 expression, which is linked to strong ligand-dependent γδTCR signaling (53), is significantly more expressed on IFNγ-committed than IL-17A-committed γδ thymocytes (51), and CD73^−^ γδ thymocytes are enriched for those undergoing type 17 differentiation in the perinatal thymus (54). Interestingly, although CD24^+^ γδ thymocytes are considered “immature,” they nonetheless express key TFs necessary for their effector acquisition, such as RORγt for Tγδ17 cells (49, 54, 55), and are surprisingly also functionally competent to produce IL-17A (51). The application of global single cell transcriptomic analysis to fetal γδ thymocytes is likely to add significant granularity to the developmental trajectories of effector programming [preprint (56)].

## Role of γδTCR

Similar to the role of TCR in αβ vs. γδ lineage choice, the γδTCR is important for determining the effector fate of γδ T cells. The current understanding supports a model with two sequential steps in commitment; first, the decision of αβ vs. γδ, and second, the decision to become an IFNγ- or IL-17A-secreting γδ T cell (3). Both steps in development are dependent on TCR signal strength integrated with numerous environmental signals. The idea that thymic selection determines the effector fate of γδ T cells was first supported by the finding that γδ T cells exposed to a TCR ligand leading to a strong TCR signal become IFNγ producers, whereas the absence of ligand or weak γδTCR signal result in the IL-17A effector fate (57). Further supporting the notion that ligand-dependent strong γδTCR signals promote the type 1 fate, DETCs, known to produce IFNγ, adopt an IL-17A producing γδ T cell fate in the absence of their selecting ligand, Skint-1 (discussed further below) (41). Conversely, enhancing γδTCR signal strength through the addition of crosslinking γδTCR antibody GL3 to fetal thymic organ cultures (FTOC) significantly reduced the number of CD44^hi^CD45RB^−^ IL-17A-committed cells while increasing type 1-associated CD44^+^CD45RB^hi^ cells (51). A similar outcome was achieved when strong TCR signals were mimicked by transduction of T cell progenitors with a constitutively active form of the kinase Lck (Lck^F505^) (49). Together, these studies suggest that the type 17 program is the default effector pathway that is otherwise repressed by strong or ligand-dependent TCR signals. Whether Tγδ17 development supported by weak TCR signaling is truly or universally ligand-independent remains to be determined.

γδ T cell effector fate choice is also influenced by specific TCR signal transduction pathways. For example, ERK signals support the type 1 program as ERK-deficient TCRβ^−/−^ mice have an increased frequency of CD27^−^ γδ T cells, and ERK-deficient KN6 γδ TCR transgenic thymocytes are skewed toward IL-17A production compared to the controls that predominately produce IFNγ (29). More recently, it was revealed that the tyrosine kinase Syk is selectively required for Tγδ17 development, through activation of the PI3K/Akt pathway downstream of γδTCR signaling (58). Studies show that impairment of TCR signal strength with SKG [Zap70 mutant (59)] and CD3DH (CD3γ and CD3δ double heterozygous) mice both have reduced frequencies of IL-17A-producing Vγ6^+^ γδT cells (60, 61). Notably, the defect in Zap70 signaling impacts Vγ4^+^ Tγδ17s as well, just to a lesser extent, while the Vγ4^+^ γδT cells in the CD3DH mice are not impaired (60, 61). These findings imply that while we group Tγδ17s into one effector class, the Vγ subsets may require specific signal strengths and downstream signaling molecules for their effector programs. Taken together, these findings also support the model that IFNγ producing γδ T cells require strong TCR signals, while IL-17A producing γδ T cells generally require weaker TCR signal strength (41, 46, 51).

## Environmental Cues

Environmental cues in the thymus are derived from both thymic epithelial cells (TECs), developing thymocytes, and other hematopoietic cells. Timing is also a critical factor, as the developmental windows in which progenitors seed the thymus influence their exposure to signals integrated from both the stromal microenvironment and resident developing thymocytes. Therefore, γδ T cell effector specialization can be influenced by various environmental cues during ontogeny.

### Lymphotoxin Signaling

**Figure 2:**
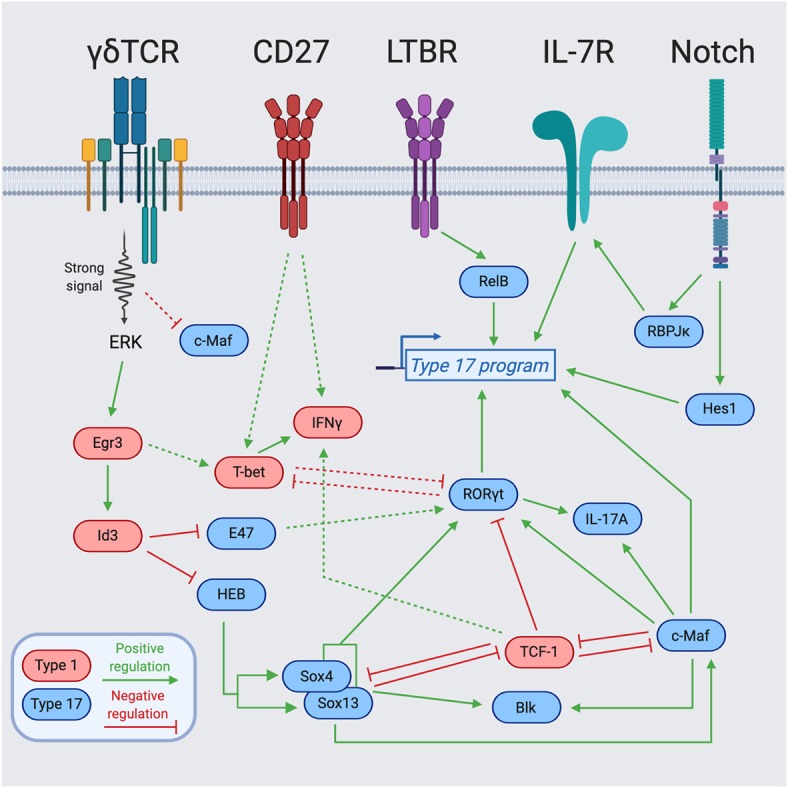
Transcription factor network regulating γδ T cell effector programming. Integration of cell surface receptors [TCR, Lymphotoxin Beta Receptor (LTBR), CD27, and Notch] with downstream transcription factors for the programming of γδ T cell effector function. Blue-colored TFs support the type 17 program, while red-colored TFs support the type 1 program. The dotted lines represent indirect regulation or that the supporting data was described in another cell type. The solid lines represent more direct regulation. Figure made with biorender.com.

One of the best-studied examples of such signals is a process called “trans-conditioning.” This phenomenon was initially discovered in TCRβ^−/−^ mice that have an altered γδ T cell gene profile and significantly reduced secretion of IFNγ by splenic γδ T cells (62). The authors concluded that αβ T cells are required for the normal development of γδ T cells (62). Subsequent work identified lymphotoxin production by DP thymocytes as the mechanism, in part, responsible for the regulation of γδ T cell maturation and differentiation toward an IFNγ-producing fate (63). Mechanistically, this was extended with the finding that CD27, a tumor necrosis factor (TNF) receptor superfamily member, engages CD70 and positively upregulates lymphotoxin beta receptor (LTBR) expression on γδ T cells (46). Accordingly, the function of CD27 in supporting IFNγ production coincides with its selective expression by mature Tγδ1 as compared to Tγδ17 cells (Figures 1, 2) (46). The role of lymphotoxin signaling in γδ T cell effector commitment is complex as the thymic differentiation of IL-17A-producing γδ T cells is also dependent on this pathway (64). Indeed, by way of the lymphotoxin signaling pathway, the NF- κb family members, RelA and RelB, play distinct roles in the thymic preprogramming of Tγδ17 cells. RelA regulates lymphotoxin ligand expression in accessory thymocytes, thereby indirectly controlling IL-17A production by γδ T cells. On the other hand, γδ T cell precursors require RelB downstream of LTBR to maintain *Rorc* expression for differentiation into mature Tγδ17 cells (Figure 2) (64). Taken together, lymphotoxin signaling regulates the effector fate acquisition of γδ T cells through integration of γδ T cell-intrinsic and extrinsic pathways.

### Cytokines and Notch Signaling

IL-7 is known for being a non-redundant, key regulator of lymphocyte homeostasis through promotion of survival and proliferation (65–68). The IL-7/IL-7R pathway plays essential roles at distinct stages in the development of multiple lymphocyte lineages (69). In particular, γδ T cells require IL-7Rα for their development, as IL-7R-deficient mice lack all γδ T cells (70). Follow-up work by several groups demonstrated that IL-7Rα-deficient mice have a block in V-J recombination of the TCRγ genes (71), and that IL-7R controls the accessibility of the TCRγ locus (72–74). While IL-7 signaling is required for all γδ T cell development, high levels of IL-7Rα expression and IL-7 signaling preferentially favor the differentiation of IL-17A-producing γδ T cells (75, 76). In line with this notion, *Aire*-deficient mice have increased production of IL-7 by medullary thymic epithelial cells (mTECs) that results in expanded populations of IL-17A-producing Vγ6^+^Vδ1^+^ T cells in the thymus and the periphery (77). The IL-7 signaling pathway also integrates with additional environmental signals and transcriptional regulators, most notably, the Notch signaling pathway. The Notch target and transcriptional repressor, Hes1, is specifically expressed in IL-17A-producing γδ T cells and Hes1 ablation significantly decreases IL-17A production with no effect on IFNγ secretion in peripheral γδ T cells (Figure 2) (78). Notch also regulates Tγδ17 differentiation in a Hes1-independent, but RBPJκ-dependent manner (79). Mechanistically, Notch signaling and RBPJκ are required for IL-7Rα expression, and IL-7Rα-mediated signaling is indispensable for the homeostasis of IL-17^+^ γδ T cells (Figure 2) (79). Future studies further exploring the transcriptional activators and repressors of *Il7r* will help elucidate how IL-7 signaling integrates with other environmental cues to control γδ T cell fate.

IL-17 is another interesting example of a soluble mediator produced in the thymus that regulates the development of γδ T cells. The development of innate-like Tγδ17 cells is restricted to a functional embryonic wave during fetal life from E16 to birth, resulting in long-lived, self-renewing cells that are found in adult mice (42). Surprisingly, it was found that IL-17 production in the thymus influences the development of Tγδ17 cells through a negative feedback loop such that CCR6^+^CD27^−^ Tγδ17 cell numbers are increased in *Il17af*^−/−^ mice (mice with deletion of the entire *Il17a* and *Il17f* locus) compared to wild-type controls (42). Interestingly, IL-17-producing Thy1^+^ cells resembling group 3 innate lymphoid cells (ILC3s) were found in the thymus of Rag1^−/−^ mice (42). Therefore, the restriction of Tγδ17 cell development may be attributed to IL-17 production from both innate lymphoid cells and IL-17^+^ αβ and γδ T cells (42).

TGF-β signaling has pleiotropic effects on immune cells. Among type 17 lineages, a specific role for TGF-β was first defined for the differentiation of naïve CD4^+^ T cells into Th17 cells. Specifically, TGF-β1^−/−^ mice have severely diminished Th17 cells in peripheral lymphoid organs (80). Despite major distinctions between Th17 cells and Tγδ17 cells, IL-17A-producing γδ T cells are also significantly reduced in mice deficient for either TGF-β1 or Smad3, the TGF-β signaling adaptor molecule, suggesting a similar dependence of TGF-β signaling for IL-17 production in the γδ lineage (81). However, this study was performed in neonates at a time point when innate-like Tγδ17 cells have left the thymus, therefore, the precise role of TGF-β signaling in Tγδ17 cell development is still unclear. In this regard, TGF-β may support Tγδ17 cells as a driver of Ras signaling (82), a signaling cascade that strongly promotes the type 17 program in γδ T cells (49).

### Butyrophilins

Whether γδ T cells undergo thymic selection analogous to αβ T cells has been a major question in the field. In order to explain the domination of tissue-specific γδ T cell compartments by particular Vγ subsets, it was hypothesized that the same γδTCR-specific ligands expressed in both the fetal thymus and target tissues could mediate positive selection during ontogeny and thereafter, tissue localization and maintenance cues for long-term residence (83). FVB-Tac mice harboring a spontaneous mutation that selectively disrupts the DETC compartment was reported to map back to a single gene expressed by TECs and keratinocytes, representing the first support for the hypothesis that DETCs undergo positive selection in the thymus (84). A few years later, the phenotype of FVB-Tac mice was attributed to a mutation in the *Skint1* gene (85). *Skint1* is a member of the butyrophilin-like (Btnl) family that structurally resembles the B7 superfamily molecules CD80 and PD-L1 (86–88). *Skint* gene expression is restricted to the thymus and skin, therefore, the broader applicability of this mechanism of selection for other intraepithelial γδ T cells was questioned (85). Recently, expression of Btnl1 by villus epithelial cells in the small intestine was shown to mediate the extrathymic selection of Vγ7^+^ intraepithelial lymphocytes (IELs), driving their expansion and maturation (89). In particular, joint expression of Btnl1 and Btnl6 by intestinal epithelial cells regulates the TCR-dependent responses of Vγ7^+^ IELs (89). Importantly, human intestinal epithelium co-expressing BTNL3 and BTNL8 selectively regulated Vγ4^+^ γδ T cells, indicating an evolutionary conserved mechanism of γδ T cell regulation across mouse and human (89). While extensive progress has been made, much remains unknown regarding the identity of γδTCR ligands that drive specific γδ T cell subset selection for tissue homeostasis (90).

### γδ T Cell Crosstalk With mTECs

Aire-expressing mTECs are necessary for central tolerance through expression of tissue-restricted antigens (91). Previous work identified the importance of RANKL-RANK signaling for induction of mTEC Aire expression by lymphoid tissue inducer (LTi) cells (92, 93). Notably, the timing of Aire expression on mTECs coincides with the first wave of Vγ5^+^ DETC precursors seeding the thymus (94). Interestingly, RANKL-RANK interactions between RANKL^+^ Vγ5^+^ DETC thymocytes and RANK^+^ mTECs also induce Aire expression and mTEC maturation. Such RANKL-RANK signaling is additionally required for Skint-1 expression by mTECs, and thus is reciprocally necessary for Vγ5^+^ DETC development. Taken together, this study elegantly demonstrates the crosstalk between developing DETC progenitors and immature mTECs that each rely on shared RANKL-RANK signals for maturation. While DETCs are the first γδ thymocytes to emerge in ontogeny, similar crosstalk between resident immune cells and TECs may account for the discrete developmental windows of other innate-like γδ T cell subsets.

## Transcriptional Networks Regulating γδT Cell Identity

γδ T cell effector acquisition is regulated by a highly-integrated network of transcriptional regulators. The lineage-defining transcription factors (LDTFs), RORγt and T-bet, promote the effector fates of IL-17A vs. IFNγ producers in various lymphocyte lineages, respectively (95–97). Although these LDTFs are integral to programming γδ T cell effector function, many other signal-dependent and collaborating TFs play essential roles in establishing and maintaining γδ T cell identity downstream of TCR signaling and various environmental signaling cascades (Figure 2).

In order to better understand the effector diversification of γδ T cells from a global perspective, the Immgen consortium performed gene-expression profiling of isolated *ex vivo* γδ T cells subsets (55). Among these, distinct clusters of immature γδ T cells could be distinguished based on their transcriptomes, reflecting three unique effector programs: IL-17A producers (Vγ6^+^ and Vγ4^+^), IFNγ producers (Vγ1^+^, Vγ1^+^Vδ6.3^+^, Vγ7^+^), and DETCs (Vγ5^+^) (55). Importantly, key TFs are enriched in specific γδ effector subsets, such as *Rorc, Maf*, *Sox13*, and *Sox4* for the IL-17A producers and *Tcf7* (TCF-1), *Lef1, Tbx21* (T-bet)*, and Eomes* for the IFNγ producers (55). The dual action of many of these TFs in both promoting one effector fate, while repressing the alternative fate leads to a complex TF network in γδ T cells (Figure 2). Interestingly, TFs associated with type 17 programming in adaptive Th17 cells—namely, IRF4, BATF, and STAT3—are dispensable for Tγδ17 cells (64, 98–100).

### TCR-Independent Transcriptional Regulators

Independent of conventional TCR signaling, innate-like γδ T cell effector programming is regulated by a quartet of HMG box TFs including Sox4, Sox13, TCF-1, and Lef1 (101). Among these, Sox13 and Sox4 are essential for the differentiation of Vγ4^+^ IL-17A-producing cells (101). This Vγ-specific requirement is intriguing as it implies that discrete regulators drive the specification of distinct subsets of Tγδ17 cells, although it remains possible that redundancy between Sox13 and Sox4 masks a global role for Sox TFs in γδ T cell type 17 programming. Within the Vγ4^+^ subset, Sox13 and Sox4 regulate key Tγδ17 program genes such as *Rorc* and *Blk* (23, 101), a tyrosine protein kinase that is selectively required for the development of Tγδ17 cells (102). While Sox proteins positively regulate type 17 fate, TCF-1 and Lef1 function to restrain Tγδ17 cell generation and gene expression (101). TCF-1 is targeted by multiple environmental signals; it is a Notch-induced TF that plays critical stage-specific roles in T cell differentiation (103, 104), and is also influenced by the Wnt signaling pathway through its β-catenin interaction domain, which is required to ensure DP thymocyte survival (104). In γδ T cells, TCF-1 promotes the expression of Lef1 and the IFNγ producing fate (101). Sox13 may also counteract the type 1 program through direct antagonism of TCF-1 via its β-catenin interaction domain (22), and indirectly via TCF-1 targets, as evidenced by Sox13 Tg mice expressing greatly diminished levels of Lef1 (101). The mutually opposing functions of Sox proteins and TCF-1/Lef1 in Tγδ1 and Tγδ17 differentiation likely reinforces and stabilizes effector fate. Together, TCR-independent HMG box TFs represent key interconnected nodes in the transcriptional network of γδ T cells.

### TCR-Dependent Transcriptional Regulators

A crucial question in γδ T cell biology is how distinct functional potentials arise from differential TCR signal strengths? (41). Broadly, effector commitment to an IFNγ-producing fate through strong TCR signaling requires both promotion of drivers of the type 1 program, and simultaneous neutralization of drivers of the type 17 program. TCR signaling can be linked to γδ T cell lineage and effector commitment through the Egr-Id3 pathway. Downstream of strong TCR signaling, Erk induced Egr1 promotes the development of γδ T cells through activation of the E protein inhibitor Id3 (26, 28). Induction of Id3 is also required for functional IFNγ production, providing a mechanism by which signal strength is translated into downstream effectors (28). This signal is key in suppression of E proteins that otherwise support Tγδ17 features (Figure 2). Indeed, it has been demonstrated in DP thymocytes that E proteins enhance RORγt expression, while Egr3 negatively regulates RORγt expression by inducing Id3 (105). Similarly, Id3 can antagonize the type 17 program by forming an inactive heterodimer with HEB, an E protein TF that is required for direct promotion of Sox13 and Sox4 expression and CD73^−^ Tγδ17 cell development (54). Along these lines, Egr3 is highly expressed in Vγ5^+^Vδ1^+^ thymocytes and upregulation of Egr3 after Skint-1-mediated selection or strong TCR signal represses *Rorc* and *Sox13* but supports *Tbx21* expression and commitment toward an IFNγ producing fate (41). Therefore, Egr3 downstream of Skint-1-mediated selection directs the TF balance necessary for proper DETC development through restraint of the “default” type 17 program. These findings highlight that TCR-dependent and TCR-independent TFs both antagonize and promote each other to regulate the effector fate of γδ T cells.

### Regulation of Type 17 Commitment

In contrast to Tγδ17 specification factors important for type 17 differentiation of distinct Vγ subsets [e.g., Sox13, Sox4, and HEB (54, 101)], the AP-1 factor c-Maf was recently identified as universally required for the generation and maintenance of all IL-17A-producing γδ T cells (49). As a canonical commitment factor, c-Maf directly activates *Rorc* and key Tγδ17 effector genes (*Il17a* and *Blk*), while also antagonizing the expression or function of negative regulators of the type 17 program (TCF-1 and Lef1) that promote the alternative Tγδ1 fate (Figure 2) (49). c-Maf globally supports a Tγδ17 chromatin accessibility landscape, with a particularly important role in the establishment of an active regulatory status at *Rorc* involving the recruitment of the histone acetyltransferase p300, and H3K27 acetylation (49). The signals that directly activate c-Maf in γδ thymocytes remain to be defined, but may involve known Tγδ17-promoting factors such as Notch, TGF-β, and IL-7 that have been described as c-Maf activators in CD4^+^ T cells or ILCs (75, 78, 79, 81, 106–108). There is some evidence that Sox TFs function upstream of c-Maf and can regulate its protein expression (49). Interestingly, unlike Sox13 expression that is independent of TCR signaling (101, 109), c-Maf expression is tuned by TCR signal strength in fetal γδ thymocytes; strong TCR signals lead to low c-Maf and weak signals result in high c-Maf protein levels, providing a mechanism by which weak γδTCR signals can be translated into Tγδ17 regulatory programming (49).

### Integration of Type 17 Regulators

A highly-integrated network of regulators control type 17 programming (Figure 2). Sox13 and Sox4 collaborate with c-Maf in the direct activation of *Rorc* and other key Tγδ17 genes such as *Blk* and *Il17a* (49, 101). The close proximity of Maf recognition element (MARE) and HMG box consensus sites in the c-Maf-dependent *Rorc* enhancer (CNS+10) suggests that c-Maf and Sox TFs may bind and function cooperatively in γδ T cells (49), as has been described in multiple other cell types (110–112). Of particular relevance, Sox5 and c-Maf can cooperatively bind the *Rorc* promoter and drive its expression in Th17 cells (112). Additionally, c-Maf and RORγt collaborate in the activation of *Il17a* and potentially other type 17 signature genes, however, c-Maf also functions independently of its direct target RORγt in regulating key Tγδ17 lineage-modulating factors (e.g., *Blk, Lef1*, and *Syk*) (49). Aside from activation of the type 17 program, both Sox13 and c-Maf repress the alternative type 1 fate by targeting TCF-1/Lef1 (49, 101). TCF-1 negatively regulates the *Rorc* locus (101), and its occupancy at *Rorc* CNS+10 is antagonized by c-Maf in γδ thymocytes (49). As TCF-1 harbors intrinsic HDAC activity (113), this antagonism may represent another mechanism by which c-Maf promotes H3K27 acetylation at the *Rorc* locus (49). Intriguingly, c-Maf also restrains the expression and function of TCF-1 in ILC3s (106), while TCF-1 represses the c-Maf/RORγt axis to limit the formation of Tc17 cells in CD8^+^ T cells (114). This suggests that c-Maf/TCF-1 antagonism is conserved across multiple lymphocyte lineages to regulate the balance of the type 1 vs. type 17 specialization.

The integration of various signals in the effector programming of γδ thymocytes suggests several tiers of regulators in specialization. In building a model, this includes: (1) specification factors (e.g., RelB, Notch, HEB, Sox13, and TCF-1) that perceive environmental signals to support type 1 or type 17 programming either universally or in the establishment of discrete Tγδ17 subsets; (2) commitment factors (e.g., c-Maf, Egr-Id3) that impart or reinforce effector identity programs, and (3) LDTFs (e.g., RORγt, T-bet) that control genes for key canonical effector functions (Figure 2). As γδ T cell selection and effector diversification occur across various DN and γδ thymocyte developmental intermediates, with numerous thymus and TCR-derived signals likely occurring over a protracted period, the temporal contributions of such inputs with respect to effector commitment remains unclear. In this regard, a recent intriguing study employing a Sox13 reporter mouse, identified DN1-like (CD117^−^CD24^+^CD25^+^) precursors in the perinatal to day 10 thymus that are prewired for the expression of the Tγδ17 gene network (e.g., *Rorc, Sox4, Tcf7, Tcf12, Maf*, *Il7r, Scart2*, and *Blk*) and are generated in a TCR-independent manner (109). Remarkably, such Sox13^+^ DN1d cells are predisposed to become CCR6^+^ IL-17A-producing cells, suggesting they are pre-committed to the Tγδ17 fate (109). Future work focused on how such effector-committed precursors intersect with the rearrangement of particular Vγ TCRs and signal strengths will broaden our understanding of the integration of environmental and TCR inputs in the effector programming of γδ thymocytes during ontogeny.

## Concluding Remarks

The last decade of research has led to enormous leaps in the understanding of tissue-resident lymphocytes, with newfound appreciation for the diversity of innate lymphocytes. Although dependent on the same LDTFs, innate-like γδ T cells and ILCs have unique transcriptional networks that control their effector fates. Such underlying distinctions in regulatory programming may translate into functional differences or non-redundant roles for innate-like γδ T cells vs. ILCs. Indeed, γδ T cells possess a TCR complex that endow them with additional environmental sensing capacities. Thus, uniquely, innate-like γδ T cell effector commitment can be controlled, in part, by the fine-tuning of key transcriptional regulators downstream of TCR signaling to both promote one fate while repressing the other. However, there is still much to be learned with respect to the establishment of transcriptional programs independent of TCR signaling and the elements that predispose γδ thymocytes to an effector fate prior to TCR expression. In the future, taking advantage of advances in single-cell sequencing and genomics techniques will lead to a higher resolution picture of γδ T cell trajectories and lineage decisions.

## Author Contributions

MP prepared and wrote the manuscript. MC edited the manuscript.

### Conflict of Interest

The authors declare that the research was conducted in the absence of any commercial or financial relationships that could be construed as a potential conflict of interest.
